# Health, human rights and freedom at stake? A critical discourse analysis of the Swedish media debate on the national COVID-19 pandemic strategy

**DOI:** 10.1080/17482631.2024.2387842

**Published:** 2024-08-08

**Authors:** Malin Hallén, Kristina Tryselius

**Affiliations:** a School of Health and Welfare, Halmstad University, Halmstad, Sweden; b Department of Health and Caring Science, Linnaeus University, Linnaeus, Sweden

**Keywords:** COVID-19, national strategy, Sweden, health, human rights, media debate, discourse

## Abstract

**Purpose:**

The aim of this study is to, in the Swedish media debate, explore the discursive constructions of challenges in human rights and freedoms following the national spatial strategy for health and survival during the COVID-19 pandemic.

**Methods:**

The study, inspired by a critical discourse analytic approach, focused particularly on the issues addressed, subject positions, relations and rhetoric. Seventeen opinion articles published in Swedish national newspapers December 2019 - February 2022 were analysed.

**Results:**

The main contents were *threats to democracy*, *threats to the freedom and rights of specific groups*, and *threats to the debate itself*. Contents were expressed through three discourse dichotomies: *contribution vs interjection, documented vs alarmistic*, and *active on the stage vs commenting from the balcony.*

**Conclusions:**

Striking about the results is the absence of dialogue, the one-way communication, and the absent politicians. It seems that the analysed debate on the challenges of the Swedish COVID-19 pandemic strategy, based on its impact on overall freedoms and rights, has not been the focus of decision-makers during the pandemic. They have neither addressed the threats highlighted in the articles, nor contributed to the discourse. This is worrying for the long-term maintenance and development of a healthy democracy.

## Introduction

The conditions for everyday life changed radically as the non-human actant SARS-CoV-2 entered the human population of the earth in the end of 2019 and rapidly spread among it, causing millions of people to develop the disease now known as COVID-19. For some, COVID-19 passed with none or only light symptoms of illness, for others it became the cause of death. States worldwide acted with both existing, as well as rather quickly invented, political means to prevent deaths and serious illness. These political strategies have challenged democratic human rights, and thereby also human health indirectly. They were largely spatial strategies, very often requesting spatial division, such as quarantines and curfews. Sweden had a unique strategy compared to other countries, not enforcing total lockdown but instead pleading for and relying on the individual citizen´s eagerness to act responsibly in relation to declared policies and guidelines (Pierre, 2020). The Swedish guidelines however, also involved spatial restrictions through e.g., requests to work from home, restrictions to travel, and keeping distance from other people. Furthermore, the guidelines emphasized not gathering and socializing publicly and privately, and staying at home when experiencing symptoms of COVID-19 (Pierre, 2020). During shorter periods activities were instructed to implement partial lockdown (Coronakommissionen, 2022a).

Formulating and implementing strategies like the ones during the pandemic are acts of biopolitics. Biopolitics “in short, refers to a series of practices and technologies established by authorities in order to protect the lives of citizens” (Marinković & Major, 2020, p. 489). The Swedish COVID-19 pandemic strategy was based upon principles of democracy and human rights as well as individual obligations, in accordance with existing national legislation, and hence operationalizing the principle of social trust. Regulations regarding spatial demarcations during the pandemic were introduced and became central in the strategy to prevent spreading of the disease. This challenged fundamental human rights, such as the right to freedom to move, hence controlled and regulated human life and conditions for health. Even though the Swedish strategy appeared liberal in an international comparison (Gustavsson & Taghizadeh, 2023; Irwin, 2020; Simons, 2020) it was nevertheless debated nationally.

How was the strategy, its effect on human rights and related health consequences discussed in Swedish media? What issues were raised? What aspects of human rights, freedom and health were addressed? And, by whom, for whom and in what way? This article will address these questions by analysing opinion articles in Swedish national newspapers. The aim of this study is thus to, in the Swedish media debate, explore the discursive constructions of challenges in human rights and freedoms following the national spatial strategy for health and survival during the COVID-19 pandemic.

### Biopolitics, health and democracy

The COVID-19 pandemic and the biopolitical measures taken in response have changed people’s lives both rapidly and exhaustively in a way never seen before (Marinković & Major, 2020). Strategies and political means to prevent death and serious illness caused by SARS-CoV-2 included restricting people’s abilities to move, and to live their lives as usual. This was an act of biopolitics as “the power over the life of the multitude […] a politics with the goal of protecting the lives of a population” (Marinković & Major, 2020, p. 490). Biopolitics carries with it a set of ideas deriving from thoughts of Foucault (2003, 2007, 2008) and later Agamben (1998) regarding political technologies, strategies, means and actions for handling a population through disciplining and managing the bodies constituting it. Historically, the biological has been dominating the social in acts of biopolitics, by promoting and protecting “bare life” (Agamben, 1998). This was also played out through the Swedish national spatial strategies during the COVID-19 pandemic when measures to prevent the SARS-CoV-2 virus from colonizing and killing the human organism was prioritized, whilst actions to promote other aspects of health, e.g., psychological and social, were downgraded. Thus, in general these actions did not align with the World Health Organization’s (World Health Organization, 1946) broad definition of health as “*a state of complete physical, mental and social well-being and not merely the absence of disease or infirmity*”, nor a salutogenic perspective on health.

The Swedish national spatial strategy for health and survival during the COVID-19 pandemic was largely developed and formulated by the Public Health Agency, upon which political decisions were made if perceived not being covered by existing legislations (Coronakommissionen, 2022a, 2022b). The civil servants at the Public Health Agency thereby got a prominent role in communicating and defending the strategy (Bauhn, 2022). However, the COVID-19 pandemic, and the corresponding biopolitics, left traces and changed several aspects of people’s everyday life on societal level (Marinković & Major, 2020). The strategies for protecting “bare life” challenged individuals and society, and they challenged, and changed public health in a more indirect, although not less appreciable way than the virus itself (Marinković & Major, 2020).

In a democratic society acting out the freedom of speech, the public media arena offers a place for open debate on urgent social issues, including matters regarding human rights closely related to democracy (Bahmueller, 2007). In the situation of the pandemic the national spatial strategy raised such issues by changing the conditions for everyday life, creating new “normalities” for individuals and society. Another crucial aspect of democracy is that power itself, politics and governmental actions can and should be debated by the people (Heysse, 2006). In the case of the COVID-19 pandemic, power played out through biopolitical actions became noticeable for the great majority of people in Sweden. To discuss and question that kind of power is according to Lorenzini (2021) necessary. To blindly accept it on the other hand, could be dangerous (Lorenzini, 2021).

### Changed geography–changed conditions for health

The freedom to move is a human right in a democratic society. It is a spatially grounded, geographical right. Biopolitics, following the above, is also geographical. The COVID-19 pandemic and related governmental actions have changed people’s geographies and the freedom to move on several scales, from the global to the “local and micro-social everyday life in which distances, spaces, breaks, and barriers are changing and being reestablished […] a geography which creates a new topography of affliction” (Marinković & Major, 2020, p. 487). Biopolitics is thus “a matter of governing mobility—and immobility” (Lorenzini, 2021, p. 43). This biopolitical governing of mobility aimed to save life and prevent serious illness among the multitude, at the same time caused ill-health conditions on social, mental, economic and physical levels through e.g., increased intimate partner violence (Gosangi et al., 2021; Lyons & Brewer, 2022), and isolation due to spatial demarcation (Clair et al., 2021; Krendl et al., 2021).

Another health aspect of the changes in geographies due to the pandemic and its related biopolitical actions has to do with health and wellbeing as an outcome of living within a democratic society that protects human rights and freedom based on the United Nations´ (UN) Universal Declaration of Human Rights (United Nations, 1948). Caring for democracy and human rights with new spatial obligations and recommendations challenged human rights and freedom and thus also basic conditions for health and wellbeing.

According to the Universal Declaration of Human Rights the right to move, and not to be prohibited to move spatially, is directly or indirectly stated in several articles. Examples of explicit formulations are found in article 3, 9 and 13 (United Nations, 1948, our italicizations).

Article 3: Everyone has the right to life, *liberty* and the security of person.

Article 9: No one shall be subjected to *arbitrary arrest, detention or exile*.

Article 13: 1. Everyone has the *right to freedom of movement and residence* within the borders of each State. 2. Everyone has *the right to leave any country*, including his own, *and to return* to his country.

Examples of more indirect wordings related to the freedom to move are found in article 12 and 27 respectively (United Nations, 1948).

Article 12: No one shall be subjected to arbitrary *interference with his privacy, family, home or correspondence*, nor to attacks upon his honour and reputation. Everyone has the right to the protection of the law against such interference or attacks.

Article 27: 1. Everyone has the *right freely to participate in the cultural life* of the community, *to enjoy the arts* and *to share in scientific advancement* and its benefits.

However, it is also stated, in Article 29 (United Nations, 1948) of the declaration that:

1. Everyone has *duties to the community* in which alone the free and full development of his personality is possible.
2. In the exercise of his rights and freedoms, everyone shall be subject *only to such limitations as are determined by law solely for the purpose of securing due recognition and respect for the rights and freedoms of others and of meeting the just requirements of morality, public order and the general welfare in a democratic society*.

Hence, according to the UN´s Universal Declaration of Human Rights, a person’s spatial freedom can be limited if considered the best for others, and for the welfare of a democratic society. With the above in mind, as well as the words of the Constitution of the World Health Organization (1946, p. 1) envisioning “the highest attainable standard of health as a fundamental right of every human being”, the importance of geographical freedom for human rights, including health, appears clearly. So, did the media debate on the COVID-19 pandemic in Sweden register this as a significant matter? With Lorenzini (2021) words about the danger of blindly accepting power in mind, a lack of public debate could be considered health threatening on a societal level. In what way and by whom were the Swedish government´s biopolitical actions during the extreme circumstances of the COVID-19 pandemic debated?

### The Swedish media debate during the pandemic

The Swedish media debate on whether nations’ chosen strategy had been the right way to go or not was characterized by fairly strong polarization. In particular, opinion journalism had a higher proportion of conflict shaping than that of general news journalism (Widholm & Mårtensson, 2021). Prominent actors in the debate were editorial writers and columnists (Ghersetti, 2021). Giritli Nygren and Olofsson (2021) draw attention to how national preventive strategies during the first phase of the pandemic, were anchored in broader public discourses (Giritli Nygren & Olofsson, 2021). In opinion and editorial articles in the largest Swedish national daily newspaper Dagens Nyheter, three crisis narratives came to dominate: health, economy and democracy. Giritli Nygren and Olofsson (2021) show that the struggle for legitimacy around the handling of COVID-19 was characterized by several conflicting perspectives or paradoxes, both within and between these crisis narratives. One interesting paradox, with relevance for the present study, reveals on the one hand a desire for increased political control to improve preparedness, management and equity of care. It calls for more visible political leadership rather than relying on the population’s compliance in adhering to restrictions. On the other hand, concerns were raised about a weakened democracy due to the stricter measures proposed to deal with Sweden’s shortcomings in pandemic management. The lack of political leadership were also juxtaposed with the prominent role of the Public Health Agency (Bauhn, 2022; Giritli Nygren & Olofsson, 2021), where the formal political leadership only marginally was involved in creating the Swedish strategy and it was unambiguously the case that the Public Health Agency was responsible for the major decisions. One possible consequence of this, as Andersson and Aylott (2020) emphasize, is that it may thus become unclear who can be held retrospectively responsible for the potential consequences of the public policy. Comparisons with Denmark (Baekkeskov et al., 2021) show significant differences in who represented the official voice. Political leaders had a much more prominent role in Denmark, while in Sweden, the expert authority was attributed the role of the main actor. In both countries, reasons and information in support of the nationally chosen strategy for health and survival were emphasized over a pluralistic discussion of possible alternative paths. Furthermore, Hanson et al. (2021) explores the interplay between national health governance, scientific advice, and media coverage in shaping the COVID-19 responses in Germany, Sweden, and the UK. Their research reveals that Sweden’s response was heavily influenced by its public health agency’s autonomous role and a high level of trust in scientific experts. In contrast, Germany and the UK displayed more direct political intervention in public health decisions. The study also highlights the media’s role in framing the pandemic response, influencing public perception and compliance with health measures. Hanson et al. (2021) conclude that the governance structures and the relationship between science and policy significantly affected each country’s approach to managing the pandemic. The criticism of Sweden’s response to the COVID-19 pandemic, particularly its deviation from more stringent lockdown measures implemented by other countries, has furthermore been analysed by Grothe-Hammer and Roth (2021). They argue that the societal backlash against Sweden’s approach reveals implicit norms about what constitutes an acceptable response to a pandemic. The authors emphasize that while death is a normal part of life, dying from COVID-19 is perceived differently due to the virus’s global impact and the moral judgements surrounding the responses to it. Their sociological analysis highlights the exceptionalism attributed to Sweden’s strategy, which diverged from the expected norm of strict lockdowns and extensive restrictions.

Sjölander-Lindqvist et al. (2020) examine the communication strategies employed in Germany, Italy, Spain, and Sweden during the early stages of the COVID-19 pandemic. Their study identifies both similarities and differences in national discourses, focusing on how governments communicated risks and mitigation strategies to the public. The authors find that while all four countries faced the challenge of balancing transparency with reassurance, Sweden’s communication stood out due to its emphasis on personal responsibility and less restrictive measures. This comparative analysis underscores the role of national context in shaping public health communication and the varying degrees of trust in governmental decisions. As Idevall Hagren and Bellander (2023, p. 17) states in a later study on the (de)legitimization of the Swedish strategy:”In order to trust authorities, citizens must be able to trust their rational, scientific grounds for decision making”.

Previous studies also highlight the multifaceted impact of social media during the COVID-19 pandemic. Social media platforms played a pivotal role in disseminating public health information and fostering connectivity, yet facilitating the spread of misinformation and conspiracy theories, thus influencing public perception and behaviours (Tsao et al., 2021). Effective communication by health authorities, characterized by transparency and proactive information dissemination, helped build public trust and mitigate misinformation (Jørgensen & Borchgrevink, 2023). Social media influencers in Sweden acted as intermediaries, making complex health information more accessible to broader audiences, including younger individuals (Olausson, 2023). Political leaders and influencers utilized Twitter to propagate specific narratives, frequently including misinformation and conspiracy theories used to delegitimize scientific advice and governmental measures (Cervi et al., 2021). These findings underscore social media’s dual role as a tool for spreading both vital information and competing narratives.

The focus of the present study on opinion pieces in traditional news media provides a rich, reliable, and influential material for examining the discourses on national COVID-19 strategies, allowing for the exploration of the complex interplay between ideas and ideologies that shape public debate.

## Methods

Fairclough’s critical discourse analysis (CDA) is a theoretical framework that draws upon the ideas from Michel Foucault for analysing the relationship between discourse, power, and ideology (Fairclough, 2012). Like Foucault, Fairclough emphasizes the importance of examining the discursive practices used to regulate and manage populations and how these practices shape our experiences, behaviours, and understandings of the world (Fairclough, 2012). However, while Foucault’s approach focuses on the broader historical, cultural, and social contexts in which discourses are produced, Fairclough’s CDA takes a more micro-level approach, focusing on the ways in which discourse is used to reproduce and maintain power relations in specific social, cultural, or historical contexts (Fairclough, 2012).

This study has been conducted inspired by a critical discourse analytic approach as it has been developed by Fairclough (1992, 1995, 1998, 2001). The approach focuses on the relationship between language and society, addressing how language is both constituted by and contributes to constituting society. Fairclough argues that discourse is “language use as social practice” and contributes to the construction of social identities, social relations and systems of knowledge and meaning (Winther Jørgensen & Phillips, 2000). Based on the aim of this study, the analytical focus has been directed towards how linguistic practices in the analysed opinion articles contribute to a specific way to talk about the issue in question. In other words, a specific way of addressing and framing challenges of human rights and health in relation to the national spatial strategies for health and survival during the COVID-19 pandemic. At the same time, it is essential to pay parallel attention to what is *not* said and what is *assumed* without being stated (Fairclough, 2001). Additionally, the analysis focuses on the social relations constructed by the language used in the articles. Here we link to the analytical concept of *subject position* (Fairclough, 1992), which is used to describe the different positions taken by the actors (i.e senders). How do subjects describe themselves, and what value and authority do they attribute to themselves? The analysis also focuses on social relations through whom the actors (claim to) represent and whom they address.

The final part of the analysis highlights aspects of rhetoric and how language is used to question and challenge. Using concepts from Fairclough’s toolbox developed for text analysis (Fairclough, 1992) and inspired by Dahlstedt and Vesterberg (2019) approach, framing and the shaping of meanings are discussed, for example through the use of tropes such as metaphors and hyperboles. Critical discourse analysis (Fairclough, 1995) provides a framework for examining how these linguistic devices contribute to the construction of social reality and the dissemination of ideologies through media narratives. Fairclough sees metaphors as tools that can both reflect and construct social realities, playing a crucial role in the discourse that shapes public opinion and policy. Metaphors, the figurative expressions used to create desirable associations beyond the literal meaning, are not neutral; they are loaded with ideological significance and can perpetuate existing power relations. Hyperbole denotes an exaggerated expression or a strong exaggeration which plays a critical role in media discourses due to its ability to amplify messages and evoke strong emotional responses. Analysing hyperbole pays attention to how the articles frame issues in order to underscore their urgency and importance.

The basis for the analysis are opinion articles in Swedish national newspapers. Opinion articles address controversial aspects of national COVID-19 strategies, offering critical discourses that challenge official narratives and highlight contentious issues. These articles can reveal underlying power structures and interests, as authors often critique governmental actions, expose policy shortcomings, and advocate for alternative approaches (Richardson, 2007; Wodak, 2015). Analysing opinion articles enables a deeper understanding of the diverse perspectives and ideological stances that characterize public discourse on national COVID-19 strategies. These articles often feature contributions from a wide array of stakeholders, including policymakers, experts, journalists, and citizens, each bringing unique viewpoints shaped by their experiences and beliefs (Entman, 2007). General news articles on the other hand, constrained by journalistic norms of objectivity and balance, may not delve as deeply into these critical and contested areas. Societal challenges and tensions might therefore not be as readily apparent in general news reporting (Entman, 2007).

The analysed opinion articles were searched online in the national digital archive for news agencies, the Media Archive (Retriever), which is Scandinavia’s largest digital archive of full text media sources. Based on the search string “debate” AND (Corona OR COVID-19)”, articles were searched in Sweden’s four largest national newspapers (Dagens Nyheter, Svenska Dagbladet, Expressen and Aftonbladet) for the period December 2019 (when reporting on Corona/COVID-19 started) to February 2022 (when the restrictions in Sweden were lifted). A manual selection was then carried out by the researchers jointly based on two questions: “Is the article an opinion piece?”, and “Does the text explicitly comment on or address human rights and health, in relation to national spatial strategies for health and survival during the COVID-19 pandemic?”. With this initial selection criterion, editorials are excluded and thus fall outside the scope of this study. Consequently, it is not the newspaper’s voice that is analysed, but rather the actors who are given space on its opinion pages. Articles responding positively to both questions were included in the analysis. The selection resulted in seventeen opinion articles where the national newspapers Dagens Nyheter and Svenska Dagbladet were represented. Expressen and Aftonbladet, both national tabloids, had no articles that met the selection questions. The analysis has focused solely on written text. Additional photographs, illustrations or tables/diagrams have not been analysed. The media material was then jointly analysed by the researchers based on the following questions:

- What is the main content of the article? What issues are raised, and in what ways are they given meaning and made comprehensible to the reader?
- Who is the sender of the article? Who does the sender(s) claim to represent? What role in the debate does the sender attribute to themselves? To whom is the article addressed?
- How is language used to address and frame the perspective and position of the author(s) of the article? How are metaphors and hyperbole used to give extended meaning to the text?

Following the analytical questions above, cross-cutting themes were identified in which content, actor(s), relations and language co-created relatively coherent discourses. All analytical work has been carried out by both researchers, initially separately and then jointly, with careful attention to validate the observations and interpretations. The analysis has been characterized by an interaction between close reading of the selected media material and an in-depth theoretical study to identify key opinions and, in parallel, to deepen both theoretical and contextual understanding.

## Results

The aim of this study was to, in the Swedish media debate, explore the discursive constructions of challenges in human rights and freedoms following the national spatial strategy for health and survival during the COVID-19 pandemic. Even though the aim mostly directed the analysis towards the “how” in the selected opinion articles, the first result expresses *what* was mainly discussed. The main contents were *threats to democracy*, *threats to the freedom and rights of specific groups*, and *threats to the debate itself*. These content elements were discursively constructed in different ways which constitutes the second result of the study, namely the three discursive dichotomies which emerged through the analysis - *contribution vs interjection, documented vs alarmistic*, and *active on the stage vs commenting from the balcony.*

### Threats to democracy, threats to freedom and rights of specific groups, and threats to the debate itself

A common theme in the analysed opinion articles was “threats”, and in particular, *threats to democracy* in general dominated. The following quotation illustrates this.

Well-intentioned democratically elected governments risk, through their own actions, to legitimise restrictions on fundamental civil rights for a long time ahead in what are becoming new dictatorships. This ultimately threatens democracy around the world. (Lindberg, 2020)

Long-term consequences versus short-term benefits for human life and health by governmental actions were thus highlighted through this theme.

Noticeable was also the recurrent theme of the *threats to the freedom and rights of specific groups* in society, whether it was children, older adults or people choosing not to get vaccinated against COVID-19. What is particularly highlighted in the analysed articles are the long-term aspects of health for specific groups when not being allowed to maintain normal social life, meeting, and moving freely. The debaters show awareness of the short-term goal of saving lives, however, emphasize the need to include and not lose sight of long-term aspects of health.

It is clear that the government’s restraining order has created an unfortunate tension with the status of the elderly as individual subjects with their own rights, friendships and family relationships. This was perhaps initially acceptable, but now four months of isolation have passed. (Melander, 2020)

Furthermore, another dominating theme in the debate was *threats to the debate itself*.

Colleagues with access to our country’s main public platforms look troubled, so preoccupied with their own fear of death that they are unable to address the issue. Or are they naive enough to think that governments in the democratic world will not take advantage of their expanded powers in the future? The unbearable lightness of naiveness is spreading in our public (Goldman, 2021).

This theme also draws attention to what is *not* present in the debate, aspects which the authors point out are significant. It is partly about how few people want, dare or are able to participate in the public discussion and partly about who is not visible at all.

### Three discourse dichotomies

Three dichotomies emerged in the critical discourse analysis when questions were posed to the articles about subject positions and language. Who was the sender? Who did the sender(s) claim to represent? What role in the debate does the sender attribute to themselves? To whom was the article addressed? And how was language, through the use of metaphors and hyperboles, used to address and frame the perspective and position of the author(s)?

#### Contribution vs interjection

A *contribution* is characterized by the sender, based on their expertise, wanting to share further knowledge and thus add something more to the ongoing debate. The characteristic of the sender is an authority, a spokesperson or otherwise expert in the subject. The sender’s text complements or nuances what has already been said, and the contribution can thus create the conditions for a deeper understanding or offer a relevant context for an event, a phenomenon or a decision. Verbally this is expressed through value free and neutral words. The sender in the example below, is a political scientist and a recognized expert on constitutional issues.

Sweden has chosen a special strategy. But this choice was not made now, in connection with the corona crisis, but almost twenty years ago. […] The new law 2004 meant a change of perspective. The possibility of limiting the spread of infection through coercive measures certainly remains. But now the single individual was also given a major responsibility for the infection control (Petersson, 2020).

Contributions have the explicit purpose of enlightening both the addressee and the general reader to bring the discussion forward, and although the language is balanced the text may include hyperboles and rhetorical questions. The sender is both speaking on behalf of the public, as well as assuming the public as the reader. However, the contributions function through their implicit addressing of the decision makers, here illustrated by an opinion article from a former Secretary of State, civil servant and business leader.

According to both the Instrument of Government and the European Convention, which is linked to the Constitution, protection of health and against epidemics are legitimate reasons for constraints, but legal restrictions on freedom of assembly and movement must be necessary, appropriate and proportionate. And since there are no infection control reasons to prevent older vaccinated people from meeting up in larger groups, what would be the reason? (Dahlsten, 2021)

An *interjection* on the other hand is characterized by the sender, an expert, authority or an affected party, engaging in a debate without substantiating what is added. The sender is first and foremost representing themselves, in the example below, concerned parents, and the interjection is addressing decision makers through texts aimed for the public reader. The interjection does not add any additional knowledge or dimension to the issue under discussion but is characterized more by the sender’s indication that they are a party to the debate.

The experience that other people do not take one’s experiences seriously can cause long-lasting reactions. Everyone must have the right to react in their own way without becoming illegal, without, of course, being allowed to neglect children’s right to education and knowledge (Almström et al., 2020).

Thus, an interjection is characterized by a kind of rhetorical underlining for the reader and addressee: “Have you seen this?”, “Have you understood this?”, and hyperboles emphasize and sharpen what is stated. This is illustrated by a quote from an article written by researchers and senior advisors in political economics and health economics.

Politicians in many countries have used the pandemic as an excuse for a more authoritarian society. In Sweden, the pandemic law leaves the field open for an arbitrary exercise of power (Jonung et al., 2021).

#### Documented vs alarmistic

The *documented* discourse is characterized by a factual and balanced way of articulating its message and the lack of strong expressions and colourful metaphors. References support the position but are not as fully developed as for a contribution.

From a child’s perspective, the aim is of course that the pandemic affects children as little as possible. This reasoning includes both direct morbidity from the virus, but also indirect effects of various restrictions. The benefits and risks in each scale must be weighed against each other. Unilateralism is irresponsible and puts children’s health at risk (Nordenhäll et al., 2021).

However, the meaning is always serious, and the senders are experts in the field they are debating, in the quote above, representatives of the Swedish Paediatric Association. There is a variety in who is addressed in the articles characterized by the documented discourse, as well as whom the sender claims to represent. In the following quote, a researcher in political history compares the Danish telesurveillance of the early 20th century with the increased surveillance in the wake of the coronavirus.

It is very difficult to roll back measures that have been introduced and with it the infringement of personal integrity by different types of surveillance. During the ongoing coronavirus pandemic, researchers have warned that decisions on increased surveillance measures risk becoming permanent after the pandemic has subsided. Based on historical experience, this is a legitimate concern (Linnarsson, 2021).

This seriousness also characterizes the otherwise contrasting *alarmistic* discourse which in addition uses strong expressions and bombastic language, often with strong and graphic metaphors.

It is sad to witness the politicians´ fundamental lack of understanding of the importance of culture for community building - in terms of democracy, health and experience. From right to left, no one today seems to have either knowledge or interest in solving the negative effects of a defunct cultural life on citizens. An active engagement in culture is much more than a pleasant decoration on the fringes of life. It is the very foundation of society. (Lindblad et al., 2020)

In this example, representatives of the academic and cultural world address the Minister of Culture directly. By taking things to the extreme the focus is on threats and intimidation that can create a strong reaction in the addressee and the reader. The *alarmistic* discourse indicates that the sender, in the quote below a federal board member of the liberal youth party, has understood something no one else has, and the language used becomes a tool to signal danger.

It is completely absurd. The right to demonstrate, freedom of assembly, to conduct business, freedom of religion, and to some extent freedom of movement are massively restricted. In anything other than a pandemic situation, Sweden would be considered a police state (Hjelm, 2021).

The alarmistic discourse does not open to further dialogue; the debate is closed by the sender’s clear statement of opinion. The senders come from many different sectors and groups in society, joined by a strong engagement in a certain aspect of life as well as in the ambition to awake and alert both public and decision makers.

#### Active on the stage vs commenting from the balcony

The discourse referred to as *active on the stage* is characterized by a sender who takes a clear place as an actor. The sender(s) are involved, often as experts in a specific field, and they take responsibility, and show through their input to the discussion that they place themselves within the current situation. The following statement from medical ethic experts illustrates this type of discourse.

We are concerned about the introduction of age as a basis for prioritization, which has been raised during the current corona crisis. […] We believe that prioritization, even under extreme conditions, should be in accordance with both the first and the second principle of the Swedish order of priorities (Skogseid et al., 2020).

When decisions regarding the national spatial strategy for health and survival are commented on, the language used is characterized by a high degree of participation. The senders show how their activities are directly affected, or they indicate a risk that someone who they represent or speak for is or might be affected negatively. Active on the stage texts lack strong or colourful metaphors although they have a straight and direct message directed towards decision makers, as can be noted in the following quote from medical experts in paediatrics.

Others know better than we do whether the spread of infection in schools is currently so high that it justifies closure to reduce social contagion and thus protect risk groups. However, suppose the issue is focused on just being about health and risk of illness of children and youth. In that case, we believe it is reasonable to keep schools and leisure activities open and accessible as far as possible (Nordenhäll et al., 2021).

The opposite discourse is called *commenting from the balcony* and is characterized by a distance where the sender assumes the position of observer and commentator. The texts evaluate and assess as the senders stand beside. The discourse is characterized by low personal responsibility and high normative statements: “Shouldn’t you…….?” or “How can they…?”.

Shouldn’t society feel a debt to the elderly who have been “stowed away” for more than a year and allow those who have been vaccinated to return to meeting physically in all the various more or less informal clubs and societies, reading circles and walking groups, that so many elderly people belong to, even if there are more than eight in the group? (Dahlsten, 2021)

The sender, as in the above, a former Secretary of State, civil servant and business leader, commenting from the balcony points a critical and blaming finger at a rather vague addressee. The language is rich in both established and innovative metaphors, used for emphasizing the identified alarming problem.

One prescribes measures that are similar to prescribing broad-spectrum antibiotics that kill every living thing in the economy, combined with extensive political radiotherapy that erodes basic civil democratic rights (Lindberg, 2020).

The quote is taken from an article written by a professor of political science. The article uses colourful language to equate measures against the spread of the virus with excessive and irresponsible medical treatment that causes more damage than the virus itself. The *active on the stage vs commenting from the balcony* dichotomy shows a clear and significant distance between the actors who take centre stage and those who adopt a distanced position.

## Discussion

The topics in the following discussion relate to the initially raised questions regarding what was discussed, how it was discussed and by whom.

### Save bare life vs long term health

The debate on the Swedish national spatial strategy for health and survival during the COVID-19 pandemic reflects an awareness of the governmental focus on saving lives, as many as possible. The debaters do not deny that this is important but add more long-term perspectives on health through their opinion articles. In recognizing that the state’s ambitions to “save bare life”, and thus achieve short-term health gains, can have negative consequences for people’s lives and health in the long term, the different discourse types are united. Through this, it becomes clear that the debate questions the implementation of a historically dominant biopolitics rather than a biopolitics based on a broader view and definition of health that pays attention to social and mental health aspects.

Fundamental spatial rights and freedoms, as expressed in the UN Declaration of Human Rights permeate the discourse by relating to several of the areas covered by the Declaration from various starting points and perspectives. A clear aspect that also emerges in the discourse, regardless of the type of discourse, is the recognition that state biopolitical measures can be introduced fairly quickly but can have both long-term health consequences and become long-term, which needs to be debated in a healthy, democratic society. In an analysis of the Swedish Corona strategy, Bauhn (2022) notes how it was characterized by utilitarianism. It was society as a system that would make it through the pandemic, and it was for the long-term survival of that system that the disease curves needed to be flattened. However, the discourses emerging in this study are not primarily characterized by the perspectives of the right to bare life, or the long-term survival of the healthcare system. Instead, attention is paid to more long-term aspects of human life and health, and the importance of considering these in a democratic society where individual, fundamental rights and freedoms are operationalized even in times of crisis.

Questions are raised about why restrictions were not lifted for everyone or for specific groups, or why certain individuals or activities were allowed in some places but not in others. What these discourses also contribute is a more nuanced and holistic perspective on health promotion work beyond merely “protecting bare life.” This perspective is perceived to be missing from the established agenda. The demand for transparency and the additional dimensions brought to the debate through various discursive patterns are important to consider, as de Campos-Rudinsky and Undurraga (2021) emphasize that transparent communication is crucial to maintain trust in authorities and decision-makers. Given that the Swedish COVID-19 strategy heavily relied on trust, it is crucial for the government and authorities to act in a trust-building and sustaining manner. This includes communicating the rationale and arguments behind decisions and being present in this media arena as well (Idevall Hagren & Bellander, 2023). This is particularly important if a similar type of trust-based strategy is to be applied in future equivalent situations.

### Discourses of great variation

The media debate on challenges arising from the national spatial strategy for health and survival in terms of its impact on human rights and freedoms, is driven by various social actors; experts in specific fields, public debaters and committed representatives of specific groups and organizations. A discursive variety characterizes the analysed articles. The debate includes both a varied use of language and a plethora of different voices, which can be seen as a positive sign of a vivid democratic dialogue. In the main, a reasonably consistent ambition to represent the interests of the public and be its voice, sometimes as part of it, sometimes as an outside observer, emerges. There is also a common direction of addressing those decision-makers by asking questions, formulating demands and critically challenging. Concerns and misgivings about choices and strategies are expressed, sometimes explicitly, with the government or responsible ministers as the explicit addressee, sometimes more vaguely addressed to “those who decide”.

Articles based on the interests and needs of specific groups tend to be more objectively expressed. This can possibly be due to the fact that the issue is closer to the sender’s specific area of expertise, and the factual arguments thus become sufficient tools in the texts. Here, colourful metaphors and hyperbole are relatively absent, and the sender, as an expert actor, is placed in contrast to the addressee, who is requested to take responsibility and act.

The opinion articles that highlight both potential threats to democracy and possible threats to the debate itself are instead characterized by descriptions rich in metaphors and hyperbole, and by that adding an extended meaning beyond the debated matter. A more aggravated use of language emerges, where the situation and the concerns raised are highlighted through descriptions of future scenarios where currently implemented or proposed limitations and restrictions have escalated and transformed into human rights-violating weapons in the service of dictatorship.

The metaphors within the alarmistic and commenting from the balcony discourses allude for example to diagnosis, illness and medical treatment and illustrate the potential or actual impact of excessive restrictions on the “health of society” and citizens’ rights and freedoms. The use of hyperbole also visualizes risk scenarios where the consequences of imposed restrictions are described as an acute and imminent threat to a democratic society.

### The present senders and the absent addressees

In Sweden, expert authorities were given a prominent role in both the design of the national COVID-19 pandemic strategy itself and its communication. They frequently expressed recommendations, advice and actions in the media and arranged press conferences. The possible long-term health consequences of the restrictions appeared to be outside the remit and responsibility of these representatives. At the same time, the discourses show a need to raise questions about the consequences of the spatial demarcations on people’s rights and freedoms and the following long-term impact on their health. In 2021, de Campos-Rudinsky and Undurraga, drew attention to the many and rapidly changing COVID-19 policies during the pandemic. They note that the great amount of data and the many decisions that were made based on it, led to an erosion in trust in public health authorities and policy since people experienced this as “erratic and at times arbitrary” made decisions, and “whimsical or defective” policies (de Campos-Rudinsky & Undurraga, 2021, p. 296). The discursive patterns identified in this study can be understood as different ways of questioning decisions and policies, and an expression of wanting to get to know the data behind, and the reasons for them. Some senders do it from the stage where the action takes place, others from the balcony they are standing on, some in a documented way, trying to fill in the knowledge gaps, and others by pressing the alarm bell. When fundamental democratic rights and freedoms are described as under threat, with following health concerns, there is no clear and present recipient in this media forum. The senders thus appear to be alone in a national democratic arena where they take on the role of promoting and representing citizens’ interests. Could this possibly reflect the unclear division of responsibilities between the expert authority and the political decision-makers in the Swedish context?

If mistrust in future healthcare decisions and public health policies is to be avoided, it appears important that health authorities and policy-makers engage in the debate in all media spaces where it plays out. This seems particularly important in the Swedish context, where the strategy during the pandemic was built on citizens´ trust and confidence (Hanson et al., 2021; Sjölander-Lindqvist et al., 2020). Upholding trust requires a reciprocal engagement, such as to partake in public debate answering questions and address concerns. Otherwise, there might be a risk that governmental and healthcare authorities´ managing of future pandemics will be mistrusted, with severe consequences for human health in both short and long-term.

Of course, it can be argued that in the acute situation there was neither time nor possibility to prioritize participation in the media debate on the possible long-term health consequences of the restrictions. In a crisis, the here and now is what counts. However, the study covers a longer period, which means that more than the acute phases of the pandemic have been included.

### Strengths and limitations of the study

The study has a focused approach on opinion articles with delimited, defined content. Therefore, the analysis has the potential to contribute to enhanced understanding within the specific area, while also offering insights into more general aspects of opinion materials in traditional news media. Through a specific focus on subject positions and relationships, the implicit or explicit claims of senders regarding their place and role in the debate are highlighted. In an extraordinary situation such as a global pandemic challenging all aspects of life and society, the study’s findings contribute to increased understanding of the dimensions, potential, and limitations of public debate in national newspapers. The study underscores the significance that addressed decision-makers are not present when urgent issues regarding human rights and freedoms are discussed.

The study limits its scope to the four largest national newspapers in Sweden. By focusing exclusively on these newspapers (Dagens Nyheter, Svenska Dagbladet, Expressen, and Aftonbladet), the analysis may therefore overlook perspectives and opinions expressed in regional or alternative media outlets. Also, the study period may not capture evolving opinions or shifts in public discourse over a longer time. For instance, opinions and debates on COVID-19 measures, vaccines, or new variants may have significantly changed between early phases and later stages of the pandemic.

The imbalance in the number of selected articles among the newspapers (with Dagens Nyheter and Svenska Dagbladet represented, but Expressen and Aftonbladet having no qualifying articles) may bias the analysis towards certain editorial policies or audience demographics. This could affect the diversity of perspectives included in the study.

The requirement that selected articles explicitly address human rights and health in relation to national spatial strategies may bias the selection and inadvertently exclude articles that discuss related topics but do not use the specified concepts. This could exclude nuanced discussions or alternative viewpoints that are crucial for a more comprehensive understanding of media discourses and how it might have influenced public opinion during the pandemic. In addition, the manual selection (initially made individually and later verified jointly) process conducted by the researchers, inevitably introduced subjectivity in deciding which articles meet the criteria.

Social media channels serve as crucial arenas for rapid and wide-ranging discussions on COVID-19, influencing public opinion and policy debates in ways distinct from traditional media. By omitting social media analysis, the study may overlook valuable insights into grassroots sentiments, community perspectives, and real-time reactions that shape broader societal attitudes towards pandemic-related issues.

## Conclusions

What is most striking about the results of this study is the absence of dialogue. The material, regardless of type of discourse, is characterized by one-way communication. Synonyms for debate include “exchange of opinions”, “discussion”, “exchange of replies”, and “exchange of views”. Is there an exchange of views when an addressee does not respond? Or do sentences and lines from different senders, expressed in different ways, just pile up? And perhaps most importantly, how healthy is such one-way “debate” in a democratic society?

Through a delimited study such as this one, focusing on the debate on challenges regarding human rights and freedom in relation to the Swedish spatial strategy for health and survival during the COVID-19 pandemic, it is possible to identify important gaps in communication. This study shows that when it comes to debating these challenges through opinion articles in national daily press, politicians were strikingly absent. Where were they? Where did they debate? And with whom? We know that there were press conferences, speeches to the nation and interviews for news programmes. There were also posts on social media such as Instagram, Twitter and Facebook. But it’s one thing to provide information on your own chosen topics, to answer questions from a group of journalists for a limited period, or to post a few sentences on a for-profit online forum. It is another thing to meet parties in debate in a forum that allows for detailed responses. It seems that the debate in the national daily press on the challenges of the Swedish COVID-19 pandemic strategy for human health and survival based on its impact on overall freedoms and rights has not been the focus of decision-makers during the pandemic.

It is possible to speculate on a variety of reasons for the absence of opinion articles signed by decision-makers in the Swedish national press on this specific issue. Perhaps there is a lack of knowledge, perhaps there is a lack of time, and perhaps it is easier to score “media points” in a populist political climate through short headline posts on social media rather than through detailed responses in the daily press. In any case, the addressed decision-makers have not taken the opportunity to participate in the debate in this national media forum and thus neither addressed the threats highlighted by the authors of the articles, nor contributed to shaping the discourse in this particular communication channel. This is worrying for the long-term maintenance and development of a healthy democracy based on social and mutual trust.
